# Colden’s Liquid Beef Tonic

**Published:** 1886-12

**Authors:** 


COLDEN'S LIQUID BEEF TONIC.—This excellent preparation has become deservedly popular with the medical profession, in the treatment of diseases where an agreeable article of diet and tonics are required. It is recommended in *typhoid and malarial fevers*, *consumption*, *loss of appetite and debility* induced by any cause, and it is tolerated when other forms of animal food are rejected. We invite those who have not used it to try it.

THE *Archives of Gynæcology, Obstetrics and Pædiatrics*, New York, series of 1886, just completed, has met with such warm encouragement, the publishers have decided to issue monthly, and, commencing January, the parts will so appear, instead of bi-monthly as heretofore.

